# Evaluation of Indolocarbazoles from *Streptomyces sanyensis* as a Novel Source of Therapeutic Agents against the Brain-Eating Amoeba *Naegleria fowleri*

**DOI:** 10.3390/microorganisms8050789

**Published:** 2020-05-25

**Authors:** Aitor Rizo-Liendo, Ines Sifaoui, Luis Cartuche, Iñigo Arberas-Jiménez, María Reyes-Batlle, José J. Fernández, José E. Piñero, Ana R. Díaz-Marrero, Jacob Lorenzo-Morales

**Affiliations:** 1 Instituto Universitario de Enfermedades Tropicales y Salud Pública de Canarias (IUETSPC), Universidad de La Laguna (ULL), Avda. Astrofísico Fco. Sánchez s/n, 38203 La Laguna, Tenerife, Spain; alu0100538700@ull.edu.es (A.R.-L.); isifaoui@ull.edu.es (I.S.); alu0101283227@ull.edu.es (I.A.-J.); mreyesba@ull.edu.es (M.R.-B.); 2 Departamento de Obstetricia, Ginecología, Pediatría, Medicina Preventiva y Salud Pública, Toxicología, Medicina Legal y Forense y Parasitología, Universidad De La Laguna, 38203 La Laguna, Tenerife, Spain; 3 Instituto Universitario de Bio-Orgánica Antonio González (IUBO AG), Universidad de La Laguna (ULL), Avda. Astrofísico Fco. Sánchez 2, 38206 La Laguna, Tenerife, Spain; lecartuche@utpl.edu.ec (L.C.); jjfercas@ull.edu.es (J.J.F.); adiazmar@ull.edu.es (A.R.D.-M.); 4 Departamento de Química y Ciencias Exactas, Sección Química Básica y Aplicada, Universidad Técnica Particular de Loja (UTPL), San Cayetano alto s/n, 1101608 Loja, Ecuador; 5 Departamento de Química Orgánica, Universidad de La Laguna (ULL), Avda. Astrofísico Fco. Sánchez s/n, 38206 La Laguna, Tenerife, Spain

**Keywords:** indolocarbazoles, staurosporine, *Naegleria fowleri*, brain, amoeba, PAM, therapy, PCD

## Abstract

*Naegleria fowleri* is an opportunistic pathogenic free-living amoeba which is able to rapidly colonize the central nervous system (CNS) and causes a lethal infection known as primary amoebic meningoencephalitis (PAM). Furthermore, more than 98% of the known cases of PAM are fatal and affect mainly children under 12 and young adults. Until now, no fully effective therapeutic agents against *N. fowleri* are available and hence the urgent need to find novel agents to treat PAM. At present, PAM therapy is based on the combination of amphotericin B, miltefosine, among others, with unwanted toxic effects. Recently, our team isolated various indolocarbazoles (ICZs) from the culture of a mangrove strain of *Streptomyces sanyensis* which showed activity against kinetoplastids and the *Acanthamoeba* genus. Hence, in this study, the activity of the previously isolated ICZs, staurosporine (STS), 7-oxostaurosporine (7OSTS), 4′-demethylamino-4′-oxostaurosporine, and streptocarbazole B, was evaluated against two type strains of *N. fowleri*. Furthermore, the performed activity assays revealed that STS was the most active ICZ presenting an inhibitory concentration 50 (IC_50_) of 0.08 ± 0.02 µM (SI 109.3). Moreover, STS induced programmed cell death (PCD) in the treated amoebae by triggering DNA condensation, mitochondrial disfunction, cell membrane disruption, and reactive oxygen species (ROS) generation. Therefore, STS could be a promising therapeutic agent against PAM.

## 1. Introduction

The free-living amoeboid species *Naegleria fowleri* is the causal agent of a fatal encephalitis known as primary amoebic meningoencephalitis (PAM) [1,2,3,4]. Furthermore, this species, also named brain-eating amoeba by the media, is able to colonize the brain after amoeboid-contaminated water enters the nasal passage of affected individuals [2,5,6]. After that, the pathogenic amoeba is able to colonize the central nervous system (CNS), causing death in more than 98% of reported cases [7,8,9]. Since 2000, the incidence of PAM has increased worldwide and it is often associated with aquatic risk activities such as swimming and diving in warm-water bodies [10].

Regarding PAM clinical symptoms, they are non-specific, and hence the diagnosis is often reached after the patients have died [11,12,13]. Apart from a late diagnosis, current therapeutic approaches are based on a combination of compounds which include miltefosine and amphotericin B as well as azithromycin and/or rifampin [14,15]. In the most recent reported cases, the addition of miltefosine in combination with hypothermia has resulted in the survival of affected patients [12,16,17]. Nevertheless, novel therapeutic molecules for PAM are needed since the current option presents undesired toxic side effects [13].

Natural compounds, especially those of marine sources, have been recently reported to present potential as antiprotozoal therapeutic agents [18,19,20,21]. Our group previously worked on elucidating the potential of natural indolocarbazoles (ICZs) isolated from the *Streptomyces sanyensis* PBLC04 strain (collected in Ecuador) as antiprotozoal agents [22,23,24]. Recently, the in vitro activity of staurosporine (STS) and other ICZs was reported against kinetoplastids and also the free-living amoeboid genus, *Acanthamoeba* [22,23]. In both cases, STS and 7-oxostaurosporine (7OSTS) molecules presented activity at low concentration, fast action, and low toxicity [22,24].

Indolocarbazoles including STS have been previously reported to present protein kinase (PK) and/or DNA topoisomerase I inhibition properties [22,25,26,27,28,29,30]. Furthermore, focusing on PK inhibitors with STS the most studied one, these enzymes are key ones in pathways of infection and survival of parasitic protozoa [31,32,33]. Moreover, STS treatment has been related to the induction of programmed cell death (PCD) or apoptotic-like processes via the interaction with PK and/or mitochondrial disruption mechanisms [22,24,34].

**Figure 1:**
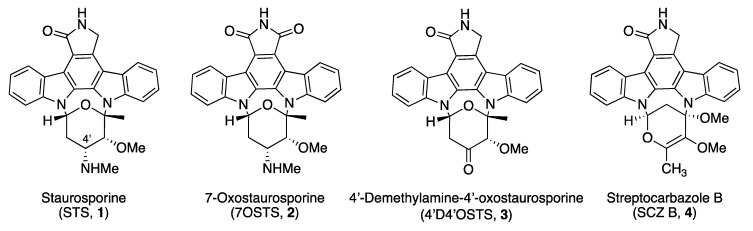
Structure of natural ICZs isolated from *Streptomyces sanyensis* PBLC04.

Since no previous reports on the activity of ICZs and natural-origin PK inhibitors has been described before for *N. fowleri* species, the aim of this study was to evaluate the therapeutic potential of some natural ICZs against this pathogenic amoeba. Hence, we aimed to analyze the anti-*Naegleria fowleri* activity of four natural ICZs previously isolated and characterized by our research team from the *S. sanyensis* PBLC04 strain collected in Ecuador (Figure 1). Moreover, elucidation of the mechanisms of induced cell death of the most active ICZ and also structure–activity relationship studies were carried out.

Our results revealed that STS statins caused induction of PCD-compatible effects in treated amoebae and hence, they could be a useful candidate for the development of novel therapeutic approaches for PAM.

## 2. Materials and Methods

### 2.1. Natural Compounds

Four indolocarbazoles previously isolated from a mangrove strain of *Streptomyces sanyensis* [22,23,24] were used in this study: staurosporine (STS, 1), 7-oxostaurosporine (7OSTS, 2), 4′-demethylamino-4′-oxostaurosporine (4′D4′OSTS, 3), and streptocarbazole B (SCZ B, 4). Stock solutions of all compounds were prepared in dimethyl sulfoxide (DMSO) and maintained at −20 °C until required. The purity and stability of each ICZ were checked by nuclear magnetic resonance (NMR) prior to carrying out the activity assays as previously described [22].

### 2.2. Chemicals

Amphotericin B (Sigma–Aldrich, Madrid, Spain) and miltefosine (Cayman Chemicals, Vitro SA, Madrid, Spain) were used as positive controls. ICZ derivatives rebeccamycin (5) (CAS no. 93908-02-2), K252a (6) (CAS no. 99533-80-9), K252b (7) (CAS no. 99570-78-2), K252c (8) (CAS no. 85753-43-1), and arcyriaflavin A (9) (CAS no. 118458-54-1, were acquired from Cayman Chemicals (Ann Arbor, MI, USA). Stock solutions of all compounds were prepared in dimethyl sulfoxide (DMSO) and maintained at −20 °C until required.

### 2.3. Amoebic Strains and Cell Line Maintenance

The American Type Culture Collection strains ATCC^®^ 30808™ and ATCC^®^ 30215™ of *Naegleria fowleri* (LG Promochem, Barcelona, Spain) were used for the activity assays. Amoebae were grown at 37 °C in axenic conditions in 2% (*w/v*) bactocasitone medium (Thermo Fisher Scientific, Madrid, Spain) supplemented with 10% (*v/v*) foetal bovine serum (FBS), 0.3 µg/mL of penicillin G and 0,5 mg/mL of streptomycin sulphate (Sigma-Aldrich, Madrid, Spain). As required by the Spanish Government biosafety guidelines for this pathogen, amoebic strains were cultured in a biological security facility level 3 at the Instituto Universitario de Enfermedades Tropicales y Salud Pública de Canarias, Universidad de La Laguna.

Evaluation of in vitro toxicity was carried out using the murine macrophage J774A.1 cell line (ATCC # TIB-67, LG Promochem, Barcelona, Spain) which was routinely cultured in Dulbecco’s Modified Eagle’s medium (DMEM, *w/v*) supplemented with 10% (*v/v*) FBS and 10 µg/mL of gentamicin (Sigma–Aldrich, Madrid, Spain), at 37 °C in a 5% CO_2_ atmosphere as previously described [35].

### 2.4. Amoebicidal Activity and Cytotoxicity Assays

#### 2.4.1. Activity Assays

The in vitro activity of the four ICZs included in this study was evaluated against trophozoites of two *Naegleria fowleri* type strains using a colorimetric assay [13]. Amoebae (2 × 10^5^ cells/mL) were placed in a sterile 96-well microtiter plate. Next, 50 μL of a serial dilution of each of the ICZs (in *N. fowleri* culture medium) was added to the plate. In all the activity assays, 2% DMSO was used to dissolve the highest dose of the molecules to prevent generation of any effects in the amoebic cells. Negative controls were also included in the assays, consisting of the addition of medium alone to the seeded trophozoites as previously described [13]. Miltefosine and amphotericin B were used as positive controls whereas the addition of medium alone to the seeded trophozoites was considered as the negative control. Lastly, 10% of the alamarBlue^®^ reagent was placed in each well, and plates were incubated with slight agitation for 48 h at 37 °C. After that, activity plates were evaluated with an EnSpire^®^ Multimode Plate Reader (Perkin Elmer, Madrid, Spain) using a wavelength of 570 nm and a reference wavelength of 630 nm.

To calculate the percentages of growth inhibition and 50% inhibitory concentrations (IC_50_), non-linear regression analyses were performed with a 95% confidence limit using the GraphPad Prism8.0.2 software program (GraphPad Software, San Diego, CA, USA). The IC_90_ values of the compounds were calculated using a GraphPad calculator (GraphPad Software, San Diego, CA, USA) based on the calculated IC_50_ and the Hill Slope (H). All experiments were performed in triplicate, and the mean values were also calculated. A paired two-tailed t-test was used for the analysis of the data, and *P* values < 0.05 were considered statistically significant as previously described [13,35].

#### 2.4.2. Cytotoxicity Assays

Cytotoxicity of the compounds was evaluated using toxicity assays established in our laboratory based on the evaluation of the toxicity induced in a murine macrophages J774.A1 cell line (ATCC^®^ TIB-67™) [35,36]. Concisely, different concentrations of the ICZs were incubated with the cell line, and alamarBlue^®^ reagent (10% of medium volume) was placed in each well and incubated for 24 h, at 37 °C under 5% CO_2_ conditions. Results were examined as described in Section 2.4.1.

### 2.5. Evaluation of Programmed Cell Death (PCD) Induction in Both Naegleria fowleri Strains

#### 2.5.1. Detection of DNA Condensation

The double-stain apoptosis detection kit constituted by Hoechst 33342 and propidium iodide (PI) (Life Technologies, Madrid. Spain) and an EVOS FL Cell Imaging System AMF4300 (Life Technologies, Madrid, Spain) were used in these experiments as per the manufacturer’s instructions. Briefly, 5 × 10^5^ cells/mL per well were incubated in a 96-well plate for 24 h at 37 °C with the previously calculated IC_90_ values of STS. The assay is able to separate three groups of cell populations: dead cells which show an intense red and low-blue fluorescence; healthy cells showing a low level of fluorescence, and cells in PCD presenting intense blue fluorescence (condensed chromatin/DNA) [37].

#### 2.5.2. Evaluation of Cellular Membrane Damage

The SYTOX Green assay (Life Technologies, Madrid, Spain) allows the detection of alterations in the permeability of the cell membrane. The SYTOX Green dye binds to DNA, showing intense green fluorescence, only if the plasmatic membrane is damaged and allows it to enter the cytoplasm [21]. Experiments were performed by incubation of 5 × 10^5^ trophozoites incubated with the earlier calculated IC_90_ of STS. After 24 h, 1 μM SYTOX Green (final concentration) was added to the cell culture and incubated for 15 min. Next, cells were examined in an EVOS FL Cell Imaging System AMF4300 (Life Technologies, Madrid, Spain).

#### 2.5.3. Assessment of Mitochondrial Function Disruption in Treated Cells

The evaluation of mitochondrial function disruption in STS-treated amoebae was carried out using the Celltiter-Glo^®^ Luminescent Cell Viability Assay (Promega Biotech Ibérica, Madrid, Spain) and the JC-1 Mitochondrial Membrane Potential Detection Kit (Cayman Chemicals, Vitro SA, Madrid, Spain). The first kit detects the levels of ATP in STS-treated cells in comparison to non-treated cells.

##### ATP Detection Levels Using the Celltiter-Glo^®^ Luminescent Cell Viability Assay

Measurement of ATP levels involved incubation of amoebae at a concentration of 5 × 10^5^ cells/mL for 24 h at 37 °C with the previously calculated IC_90_ of STS. Finally, the data were obtained using an EnSpire^®^ Multimode Plate Reader (Perkin Elmer, Madrid, Spain) as described before [21].

##### JC-1 Assay for the Detection of Alteration in the Mitochondrial Membrane Potential

Mitochondrial Membrane Potential evaluation was started by using a stock solution of 5 × 10^5^ cells/mL which were incubated in a 96-well plate with the IC_90_ of STS for 24 h at 37 °C. Images were captured using an EVOS FL Cell Imaging System AMF4300 (Life Technologies, Madrid, Spain). The obtained staining pattern permitted the identification of two groups in a cellular population: live cells which would show only red fluorescence and cells presenting low mitochondrial membrane potential (undergoing PCD) which would show an intense level of green and red fluorescence [21].

### 2.6. Generation of Intracellular Reactive Oxygen Species (ROS)

The CellROX Deep Red fluorescent assay (Thermo Fisher Scientific, Madrid, Spain) was used to evaluate the generation of intracellular reactive oxygen species (ROS) in STS-treated amoebae. Experiments were carried out after incubation of 5 × 10^5^ cells/mL with STS for 24 h at 37 °C. Next, cells were exposed to 5 μM CellROX Deep Red for 30 min at 37 °C in the dark and observed after using the EVOS FL Cell Imaging System AMF4300 (Life Technologies, Madrid, Spain). The presence of intracellular ROS was shown by the detection of high red fluorescence in the cells [21].

## 3. Results

### 3.1. In Vitro Activity and Toxicity of the Tested Natural ICZs against Naegleria fowleri

Activity assays revealed that staurosporine (STS, 1) and 7-oxostaurosporine (7OSTS, 2) were the most active tested ICZs against both strains of *N. fowleri* as shown in Table 1. In contrast, 4′-demethylamino-4′-oxostaurosporine (4′D4′OSTS, 3) and streptocarbazole B (SCZ B, 4) were only active against the *N. fowleri* ATCC 30808 strain. Furthermore, STS (1) and 7OSTS (2) showed IC_50_ values ranging from 0.08 ± 0.02 to 1.28 ± 0.35 µM for both tested strains. Therefore, ICZs 1 and 2 were more active than the currently used compounds miltefosine (from 38.74 ± 4.23 to 81.57 ± 7.23 µM depending on the strain, Table 1) and amphotericin B (from 0.12 ± 0.03 to 0.16 ± 0.02 µM, differing on the strain assayed, Table 1).

Furthermore, STS (1) and 7OSTS (2) showed lower values of cytotoxicity than miltefosine after performance of toxicity assays (Table 1). In the case of amphotericin B, STS (1) showed similar levels of toxicity when compared to the reference drug. Hence, STS was selected as the candidate for the evaluation of PCD induction in treated cells due low IC_50_ and toxicity.

### 3.2. Evaluation of PCD Induction in STS-Treated Amoebae

**Figure 2:**
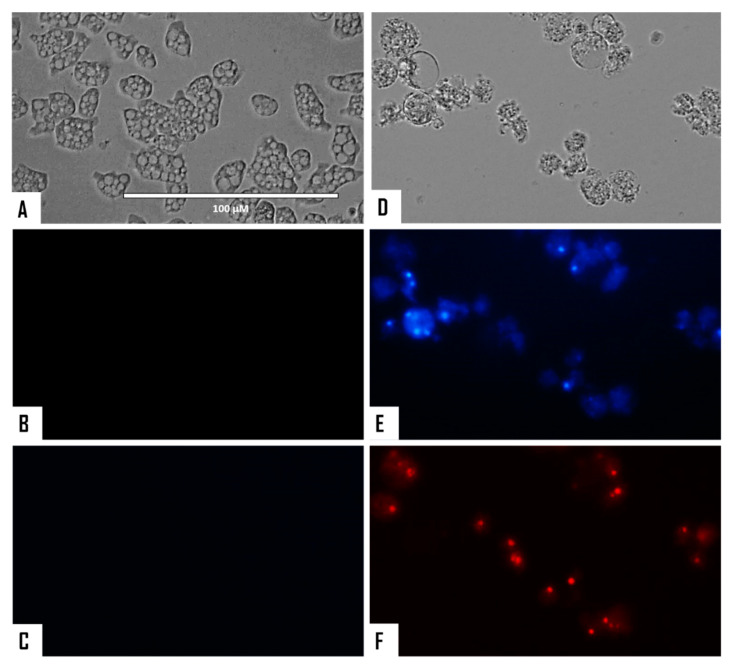
*Naegleria fowleri* trophozoites incubated with IC_90_ of STS for 24 h (**D**–**F**). Negative control (**A**–**C**). Hoechst stain is different in control cells, where no fluorescence is observed (**B**), and in treated cells, where the nuclei are intense blue (**E**). Red fluorescence corresponds to the propidium iodide stain (**C**,**F**). Visible channel (**A**,**D**); Hoechst channel (**B**,**E**), and propidium iodide channel (**C**,**F**). Images (40×) are representative of the cell population observed in the performed experiments. Images were obtained using an EVOS FL Cell Imaging System AMF4300, Life Technologies, USA. Obtained results were similar for both *N. fowleri* type strains.

**Figure 3:**
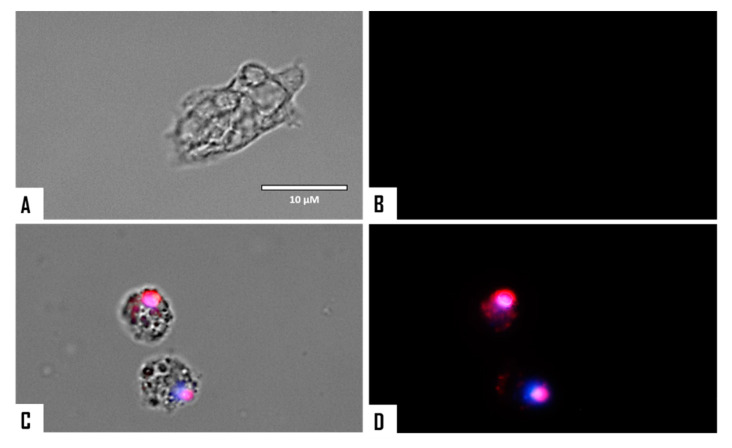
Merged and higher magnification of Figure 2. Negative control (**A**,**B**). *Naegleria fowleri* trophozoites incubated with IC_90_ of STS for 24 h (**C**,**D**). Images (100×) show late apoptotic cells with intense blue and red fluorescence. Images are representative of the cell population observed in the performed experiments. Images were obtained using an EVOS FL Cell Imaging System AMF4300, Life Technologies, USA. Obtained results were similar for both *N. fowleri* type strains.

The chromatin condensation assay was positive after observation of IC_90_-treated *N. fowleri* cells (treatment was conducted for 24 h). Treated amoebae exhibited the characteristic intense blue fluorescent-stained nuclei as shown in Figure 2 and Figure 3, which is caused by condensed chromatin.

**Figure 4:**
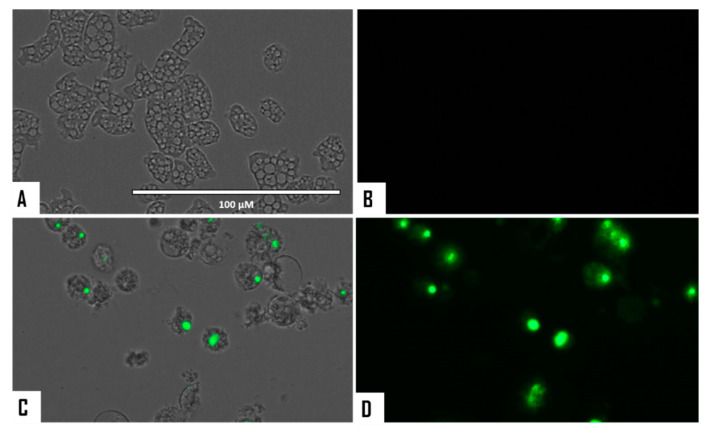
Permeabilization of the *Naegleria fowleri* plasmatic membrane to the vital dye SYTOX^®^ green caused by incubation with the IC_90_ of STS for 24 h (**C**,**D**). Negative control (**A**,**B**). Images (40×) are representative of the cell population observed in the performed experiments. Images were obtained using an EVOS FL Cell Imaging System AMF4300, Life Technologies, USA. Obtained results were similar for both *N. fowleri* type strains.

**Figure 5:**
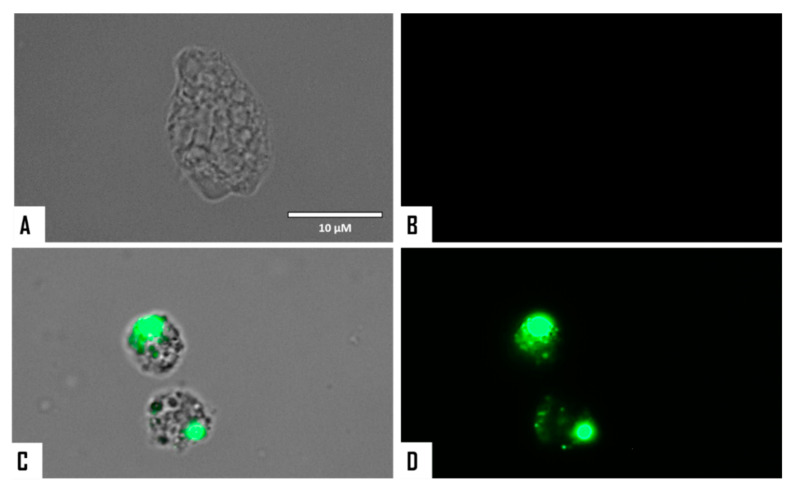
Higher magnification of Figure 4 (100×). Negative control (**A**,**B**). Cells treated with the IC_90_ of STS for 24 h (**C**,**D**). Images were obtained using an EVOS FL Cell Imaging System AMF4300, Life Technologies, USA. Obtained results were similar for both *N. fowleri* type strains.

**Figure 6:**
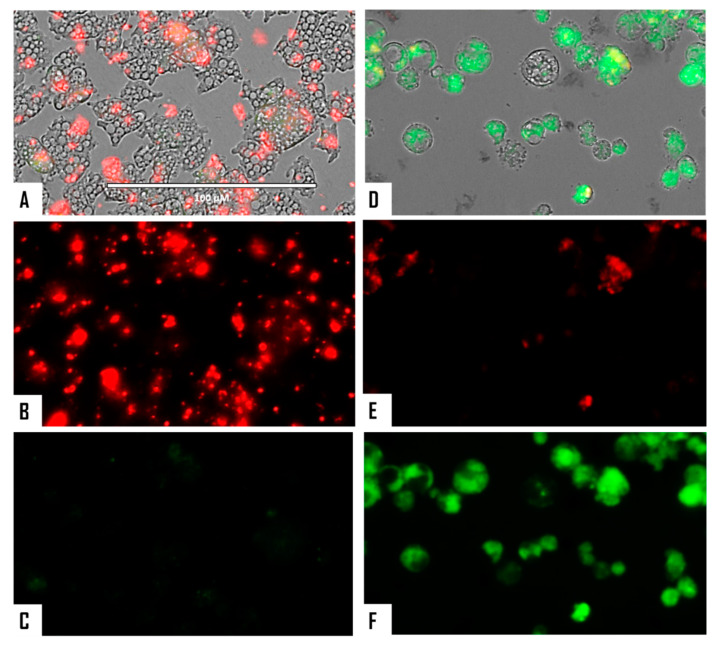
Effect of STS (1) on the mitochondrial membrane potential; the JC-1 dye accumulates in the mitochondria of healthy cells as aggregates and emits a red fluorescence. Negative control (**A**–**C**). Cells treated with the IC_90_ of STS for 24 h emitted a green fluorescence (**D**–**F**); due to the decrease in mitochondrial membrane potential, the JC-1 dye remained in the cytoplasm in its monomeric form and emitted in this case, a green fluorescence. (Images are representative of the population of treated cells, 40×). Images were obtained using an EVOS FL Cell Imaging System AMF4300, Life Technologies, USA. Obtained results were similar for both *N. fowleri* type strains.

**Figure 7:**
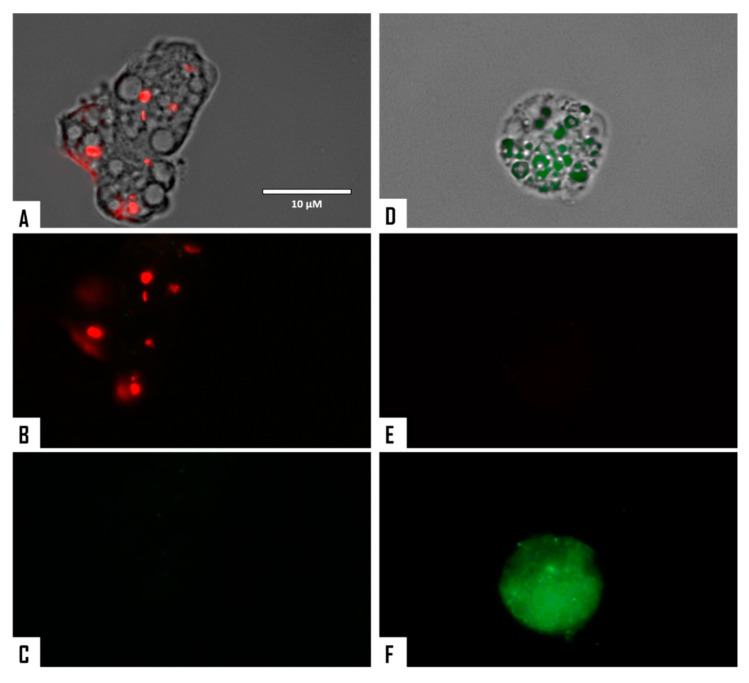
Higher magnification of Figure 6 (100×). Images were obtained using an EVOS FL Cell Imaging System AMF4300, Life Technologies, USA. Negative control (**A**–**C**). Treated cells with STS (**D**–**F**). Obtained results were similar for both *N. fowleri* type strains.

**Figure 8:**
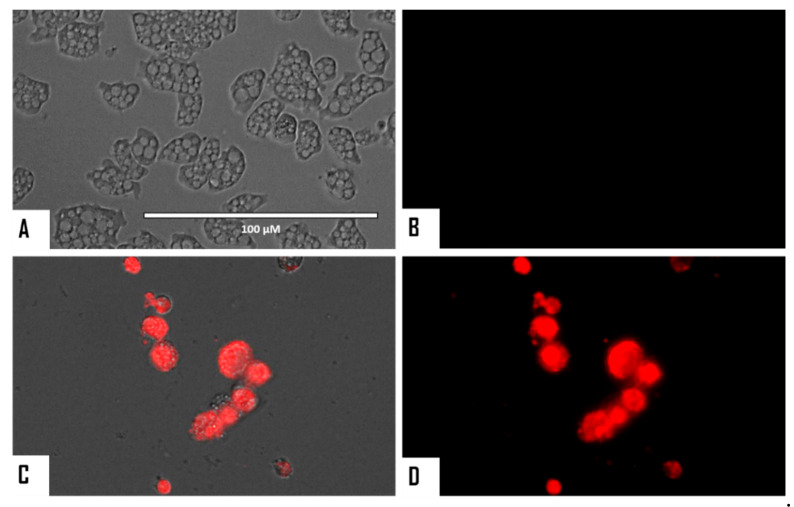
Increased levels of intracellular ROS after 24 h in *Naegleria fowleri* caused by the addition of STS at IC_90_ (**C**,**D**). STS was added to the cells (10^5^ cells/mL) and exposed to CellROX^®^ Deep Red (5 μM, 30 min) at 37 °C in the dark. Negative control (**A**,**B**): cells exposed to CellROX^®^ Deep Red in the absence of STS. Images (40×) are representative of the cell population observed in the performed experiments. Images were obtained using an EVOS FL Cell Imaging System AMF4300, Life Technologies, USA. Obtained results were similar for both *N. fowleri* type strains.

**Figure 9:**
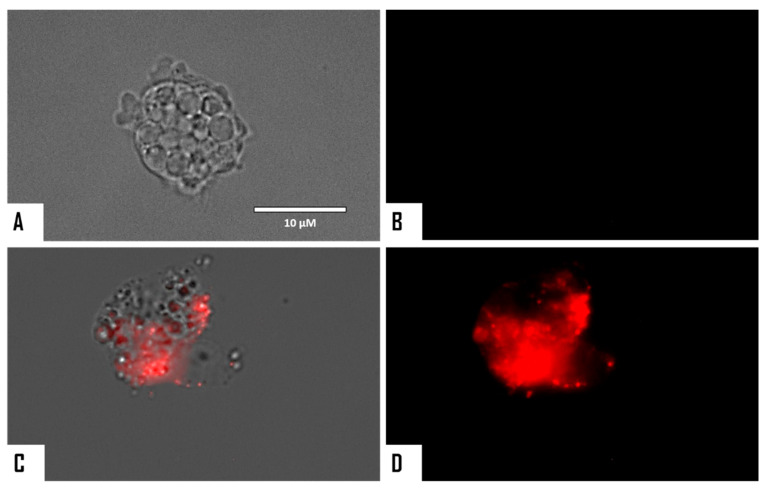
Higher magnification of Figure 8 (100×). Negative control (**A**,**B**). Cells treated with the IC_90_ of STS for 24 h (**C**,**D**). Images were obtained using an EVOS FL Cell Imaging System AMF4300, Life Technologies, USA. Obtained results were similar for both *N. fowleri* type strains.

When cellular membrane damage was observed in STS-treated amoebae (SYTOX Green Assay), an intense green fluorescence was observed (Figure 4 and Figure 5), and thus STS caused plasmatic membrane permeabilization in both strains of *N. fowleri*. Mitochondrial potential collapse was observed after treatment of cells with the IC_90_ of STS which showed green fluorescence (JC-1 in monomeric form, Figure 6 and Figure 7). The generation of intracellular ROS was shown in treated amoebae as an intense red fluorescence as shown in Figure 8 and Figure 9. 

## 4. Discussion

Indolocarbazoles (ICZs) have been intensely studied due to their inhibition properties and potential as chemotherapeutic agents and some of them have even reached clinical trial stages [38,39]. Moreover, these molecules are classified based on the targeted protein and hence are subdivided into (a) protein kinase and (b) DNA topoisomerase I inhibitors [40,41].

In this study, we aimed to evaluate the potential activity of four natural ICZs (1–4) previously isolated from the culture of the *S. sanyensis* PBLC04 strain which was collected from a mangrove in Ecuador [22,23,24]. All the mentioned compounds are reported PK inhibitors and have shown activity against other parasitic protozoa such as various species of *Leishmania*, *Trypanosoma cruzi*, and the *Acanthamoeba* genus [22,23,27,32]. Furthermore, activity assays revealed that STS (1) and 7OSTS (2) were the most active molecules, presenting IC_50_ values ranging from 0.08 ± 0.02 to 1.28 ± 0.35 µM, against both strains of *N. fowleri*, and lower levels of cytotoxicity than miltefosine after performance of toxicity assays (Table 1). In addition, the two other evaluated ICZs were only active against the type strain ATCC 30808 which could be due to long-term culture of this strain in the laboratory (higher sensitivity to compounds) [42,43,44] and/or to differences in the PK profiles of both strains [45,46,47].

Previous studies have reported that *N. fowleri* presents several PKs related to important cell roles such as fibronectin interaction, differentiation, and survival [46,47], as well as protection of *N. fowleri* amoeba against complement-mediated lysis [46]. Moreover, around 250 PKs have been detected in the complete genome project of the species *N. gruberi* [48], and hence, their potential as druggable therapeutic targets against PAM has been previously discussed [49]. Therefore, inhibition of these kinases could lead to induction of cell death mechanisms which were evaluated in our study using STS, the most active compound among the tested ICZs.

Programmed Cell Death (PCD) involves multiple cellular changes such as chromatin condensation, cell shrinkage, and loss of mitochondrial potential, among other phenomena [21,22,50,51,52]. In the present work, STS-treated amoeba presented cellular membrane damage, chromatin condensation, collapse of mitochondrial function as well as increased levels of intracellular ROS. Furthermore, effects at the cellular level were even observed after 30 min of treatment with STS. Based on the observed events, it is hypothesized that STS induces PCD through the intrinsic pathway due to the dramatic levels of mitochondrial function collapse which was observed. Moreover, no significant differences were observed in the activities and triggered mechanisms between the two strains of *N. fowleri* included in the study; hence the importance of STS as a novel therapeutic agent, since it could be effective against a wide range of *N. fowleri* strains/isolates.

Although blood brain barrier permeability could be an issue for PAM therapy, and the ability of ICZs to penetrate this barrier should be assayed, available data and modifications of ICZs to improve permeability should be the way forward [53]. Moreover, heterocyclic scaffolds, such as those based on the indole, carbazole, and indolocarbazole ones, have been highlighted as promising anticancer agents against tumours of the CNS. In addition, no clinical trials are available for PAM due to the rare frequency of these infections. As a consequence, most of the available data regarding drug efficacy are based on in vitro studies and/or clinical case reports [14]. Undoubtedly, future in vivo studies using STS in a PAM model should be performed in the near future.

Previously reported interaction studies of ICZs and PKs [39,41] have established a critical hydrogen bond interaction between the heteroatoms of the lactam moiety of STS with a conserved glutamic residue at the protein active site [22]. In addition, in closely STS-related compounds, the methylamino group at C-4′ is involved in the formation of two hydrogen bonds with amino acids (Glu, Asp) involved in the catalytic pocket. Furthermore, as previously reported [22], these interactions fix a boat-type conformation of the sugar moiety, which is perpendicularly located to the planar sp2 ICZ fragment and is related to their inhibitory activity of PKs. Hence, those ICZs exhibiting functionalization at the carbon C-4′ present an increased activity in the function of the number of hydrogen bonds between the nitrogen at the methyl amino moiety of neighboring protein residues [22].

Therefore, and based on this model, STS (1) and 7OSTS (2) (assuming that STS is the leader compound) presented similar activities even though STS was much more active against both *N. fowleri* strains. These compounds present the lactam group and the methyl amine at the C-4′ position with similar orientation and conformation (Figure 1). Thus, both compounds interact with the conserved aminoacidic residues in amoebic PKs and hence show similar activities. These observations are also supported by the fact that similar results have been reported in other parasitic protozoa [22,23]. In addition, the two others tested ICZs which differ from STS (1) and 7OSTS (2) in the sugar moiety were less active and only affected the viability of one of the strains (Table 1).

**Figure 10:**
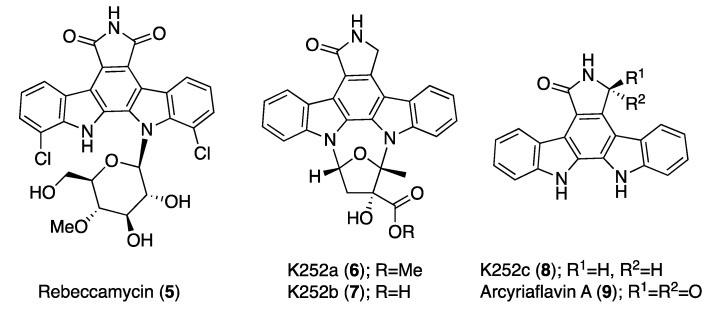
Structure of commercially available ICZs (**5**–**9**).

In order to provide an initial structure-activity relationship (SAR), the commercial ICZs rebeccamycin (**5**), K252a (**6**), K252b (**7**), K252c (**8**), and arcyriaflavin A **(9**) were also evaluated (Figure 10 and Table 2).

Among these compounds, only K252a (**6**) showed activity ranging from 0.118 ± 0.026 (ATCC 30215) to 0.338 ± 0.090 (ATCC 30808). K252a (**6**) is a known cell-permeable PKA and PKC inhibitor, and consequently, differences in the activities against the tested strains could be due to variation among their profiles of PKA and PKC. Thus, natural and commercial ICZs with log *P* values between 3.60 and 3.90 are those that show the highest of activity. Finally, rebeccamycin (5), a known DNA topoisomerase-I inhibitor, was not active against the pathogenic amoebae, supporting that the tested natural ICZs affect the viability of *Naegleria fowleri* via PKs. In the case of the respective aglycones of STS and 7OSTS, K252c (**8**), and arcyriaflavin A (**9**), no activity was observed (Table 2), confirming that the sugar moiety is highly relevant for the inhibition process of the amoebic PKs as previously reported [22,23].

## 5. Conclusions

The evaluation of the activity of STS (1) revealed high activity against the trophozoite stage of *Naegleria fowleri*. Moreover, a minor ICZ isolated from the *S. sanyensis* strain used as a source in this study, 7OSTS (2), also showed high activity values but was excluded from further assays due to higher toxicity. Nevertheless, both compounds presented high activities against *N. fowleri* which is due to the similar structure and conformation features of these compounds which interact in a similar orientation with the aminoacidic residues of the amoebic PKs. Moreover, STS induced PCD in treated amoebae via the mitochondrial (intrinsic) pathway. Therefore, STS is presented as a highly active PCD inducer and low-toxic anti-*Naegleria fowleri* compound which could be considered for future PAM therapy.

## Figures and Tables

**Table 1:** Inhibitory concentrations (IC_50_) of natural ICZs (1-4) evaluated against the trophozoite stage of *Naegleria fowleri* type strains ATCC^®^ 30808™ and ATCC^®^ 30215™ and toxicity values (CC_50_) against murine macrophage J774A.1. NA indicates that no activity was observed. (Mean concentration ± standard deviation (SD))**.**

Compound	*N. fowleri* ATCC^®^ 30808™ IC_50_ (µM)	*N. fowleri* ATCC^®^ 30215™ IC_50_ (µM)	Murine Macrophage J774.A1 CC_50_ (µM)	SI * (CC_50_/IC_50_)	Log P **
STS (**1**)	0.08 *** ± 0.01	0.08 **** ± 0.02	8.74 ± 0.72	109.3	3.86
7OSTS (**2**)	1.28 ± 0.35	1.07 ± 0.23	5.20 ± 1.75	4.1	3.74
4′D4′OSTS (**3**)	10.79 ± 2.92	NA	>20	>1.9	3.38
SCZ B (**4**)	11.51 ± 0.31	NA	>20	>1.7	3.43
Amphotericin B	0.12 ± 0.03	0.16 ± 0.02	≥200	≥1666.7	-
Miltefosine	38.74 ± 4.23	81.57 ± 7.23	127.88 ± 8.85	3.3	-

* Refers to *Naegleria fowleri* ATCC^®^ 30808™; ** Calculated with ChemDraw (version 19.1.0.5), PerkinElmer Informatics, Inc.; *** IC_90_ = 0.58 ± 0.16; **** IC_90_ = 0.97 ± 0.14.

**Table 2:** Inhibitory concentrations (IC_50_) of the commercial ICZs (**5**–**9**) evaluated against the trophozoite stage of *Naegleria fowleri* type strains ATCC^®^ 30808™ and ATCC^®^ 30215™ and toxicity values (CC_50_) against murine macrophage J774A.1. NA indicates that no activity was observed. (Mean concentration ± SD).

Compound	*N. fowleri* ATCC^®^ 30808™ IC_50_ (µM)	*N. fowleri* ATCC^®^ 30215™ IC_50_ (µM)	Murine Macrophage J774.A1 CC_50_ (µM)	SI * (CC_50_/IC_50_)	Log P **
Rebeccamycin (**5**)	NA at 20	-	1.42 ± 0.19	-	2.57
K252a (**6**)	0.34 ± 0.09	0.12 ± 0.03	1.07 ± 0.21	3.2	3.66
K252b (**7**)	NA at 20	-	≥20	-	3.39
K252c (**8**)	NA at 20	-	35.40 ± 2.47	-	3.09
Arcyriflavin A (**9**)	NA at 20	-	≥20	-	2.96

* Refers to *Naegleria fowleri* ATCC^®^ 30808™; ** Calculated with ChemDraw (version 19.1.0.5), PerkinElmer Informatics, Inc.
